# Daily emotion contagion in parent–adolescent dyads: Variations by adolescent suicidal ideation

**DOI:** 10.1111/jora.70248

**Published:** 2026-08-13

**Authors:** Shou‐Chun Chiang

**Affiliations:** ^1^ Department of Educational Psychology Texas A&M University College Station Texas USA

**Keywords:** adolescent emotion, daily diary, emotion contagion, suicidal ideation

## Abstract

Emotion contagion within parent–adolescent dyads represents a fundamental mechanism through which daily family interactions shape adolescent emotional well‐being. Yet little is known about how adolescent suicidal ideation reorganizes daily patterns of emotional transmission between parents and adolescents. The present study examined daily parent–adolescent emotion contagion and the role of adolescent suicidal ideation using a 14‐day daily diary design and a multi‐group actor–partner interdependence modeling framework. Participants were 156 adolescent–parent dyads (adolescents: M = 14.66 years, SD = 1.47; 58% female) recruited from communities in Texas. Results showed that limited emotion contagion between parents and adolescents was observed in families of adolescents without suicidal ideation. In families with adolescent suicidal ideation, two novel prospective partner effects emerged: parent positive emotions predicted lower adolescent positive emotions the following day, and adolescent positive emotions predicted lower parent positive emotions the following day. These findings indicate that adolescent suicidal ideation may characterize daily parent–adolescent emotion contagion processes, such that the presence of suicidal ideation is associated with distinct patterns of dyadic emotional transmission compared to families without suicidal ideation.

## INTRODUCTION

Adolescence is a pivotal period for the development of emotion regulation, during which emotional experiences become more intense, variable, and interpersonally consequential (Bailen et al., 2019; Silvers, 2022). Although regulatory capacities expand considerably during this period, adolescents remain deeply embedded in the family context, where parents' emotions continue to shape youth's day‐to‐day affective experiences through a process of emotion coregulation (Butler & Randall, 2013; Cole et al., 2019; Morris et al., 2007). A central mechanism through which this coregulatory influence operates is emotion contagion, whereby parental emotional states spread to and alter adolescent emotions in the course of proximal daily interactions (Hatfield et al., 1993; Lougheed, 2020). Understanding how this contagion process unfolds in daily life, and for whom it is most consequential, is critical for identifying which adolescents are most vulnerable to family emotional transmission and why.

Prior work has demonstrated that parent‐to‐adolescent emotion contagion occurs at the daily level and varies in strength as a function of relationship quality (Chiang, 2025). However, limited research has examined whether youth suicidal ideation may shape the associations between parent and adolescent emotions. Adolescent suicidal ideation may not only alter adolescents' sensitivity to parental emotional inputs but may also reorganize parental emotional experiences, introducing bidirectional processes of altered sensitivity and dysregulation into the dyadic system (Chiang & Bai, 2022; Cox & Paley, 1997). The present study addresses these gaps by examining daily parent‐to‐adolescent emotion contagion for both positive and negative emotions among adolescents with and without suicidal ideation, using a daily diary design that captures within‐person fluctuations across consecutive days.

### Theoretical frameworks of parent–adolescent emotion contagion

Emotion contagion refers to the process through which one person's emotional state is related to emotional experiences of a proximal other, broadly defined as the process by which the emotions of one individual become more similar to those of another as a result of exposure to those emotions, operating through both conscious and unconscious pathways (Goldenberg & Gross, 2020). This process has been described in the literature as emotional transmission, spillover, or dyadic emotion coregulation (Butler & Randall, 2013; Larson & Gillman, 1999; Lougheed, 2020). Despite these differences in terminology, they share a common recognition that emotions are not solely intrapersonal phenomena but are fundamentally shaped by the relational contexts in which individuals are embedded.

Within the family, this process is theoretically grounded in the tripartite model of family influence on child emotion regulation (Morris et al., 2007), which identifies three interrelated pathways through which the family context shapes youth emotional development: observational learning from parental emotional displays, direct parenting practices including emotion coaching and socialization, and the broader emotional climate of the family (Morris et al., 2018). Central to the tripartite model is the mechanism that adolescents observe and internalize parents' emotional experiences, such that parental affective states become a primary input into adolescents' own regulatory processes. Extending this framework, the Temporal Interpersonal Emotion Systems (TIES) model (Butler, 2011) conceptualizes parent–adolescent dyads as dynamic emotional systems in which the emotional states of each partner are temporally coupled across successive interactions. TIES emphasizes that emotion contagion is not a static trait‐level tendency but a time‐varying process that unfolds moment to moment and day to day, making intensive longitudinal designs particularly well‐suited for capturing its operation in daily family life.

Empirical research informed by these frameworks has documented associations between parent and adolescent emotional experiences, with early work focusing on negative emotion contagion. Studies consistently show that, on days when parents report higher levels of negative affect, adolescents tend to report elevated negative emotions as well, reflecting dynamic within‐person processes of daily emotional exchange (Larson & Gillman, 1999; LoBraico et al., 2020; Moed et al., 2017). More recently, daily diary studies have expanded this focus to examine both positive and negative emotion contagion simultaneously, yet findings remain inconsistent. For instance, one daily study found that parent and adolescent happiness were significantly linked whereas daily distress levels were not associated (Mercado et al., 2019), while another found that higher daily parent negative emotions were associated with greater adolescent negative emotions but no evidence of positive emotion contagion emerged (Chiang, 2025). Laboratory‐based research further demonstrated that maternal negative emotions during problem‐solving discussion were associated with changes in adolescent negative emotions within the dyad, but their positive emotions were not correlated (Mancini et al., 2016). These inconsistent results warrant the need for investigating daily parent–adolescent emotion contagion, especially considering both same‐valence (e.g., positive‐to‐positive emotion contagion) and cross‐valence (e.g., positive‐to‐negative emotion contagion) processes. As a result, our understanding of valence‐specific emotion contagion remains scarce in literature.

An additional gap concerns the temporal structure of emotion contagion models. The majority of prior studies have examined concurrent associations between parent and adolescent emotions, without accounting for each partner's emotional state at prior time points. This approach conflates within‐day emotional covariation with prospective emotional transmission, making it difficult to determine whether one partner's emotions are genuinely predicting changes in the other partner's emotions over time or simply reflecting shared emotional experiences within the same day. Modeling next‐day emotions as outcomes while considering same‐day emotional states allows for a more rigorous test of whether parental emotions prospectively predict adolescent emotional experiences, and vice versa, beyond what would be expected based on their concurrent emotional states alone. Together, the present study aims to clarify the mixed findings in the emotion contagion literature by examining both same‐valence and cross‐valence emotion contagion processes in daily family life, while addressing the temporal gap by modeling next‐day emotions as outcomes and accounting for prior emotional states within the dyad.

### The role of youth suicidal ideation in emotion contagion

Adolescent suicidal ideation is rooted in interpersonal experience. According to the interpersonal theory of suicide (Joiner, 2005; van Orden et al., 2010), suicidal ideation emerges from the co‐occurrence of thwarted belongingness, characterized by a perceived absence of meaningful social connection, and perceived burdensomeness, the belief that one constitutes a liability to significant others. Both of these interpersonal experiences are inherently relational and are likely to heighten adolescents' vigilance toward the emotional states of those around them (Chiang et al., 2024). Adolescents who perceive themselves as disconnected from or burdensome to their parents may be particularly attuned to parental emotional cues, as these cues carry direct implications for their appraisals of relational security and self‐worth within the family (e.g., Hunt et al., 2022; Timmons et al., 2011). From this perspective, families with and without adolescent suicidal ideation may demonstrate qualitatively distinct patterns of daily emotion contagion, as the interpersonal context created by suicidal ideation may render day‐to‐day parental emotional experiences more consequential for adolescent affect. This conceptualization is consistent with stress sensitization frameworks that document that current psychological states, even when not chronic or trait‐like, can meaningfully reorganize interpersonal sensitivity and emotional reactivity within close relationships (Chiang et al., 2023; Monroe & Harkness, 2005).

This hypothesis may be supported by the Three‐Step Theory (Klonsky et al., 2021; Klonsky & May, 2015), which posits that connectedness serves as a critical buffer against the escalation of suicidal ideation in the context of psychological pain. When connectedness is experienced as low or threatened, individuals become more susceptible to interpersonal emotional inputs that may compound their distress (Tsai et al., 2021; Yang et al., 2019). Furthermore, when emotional connectedness between parents and adolescents was high, it may attenuate the impact of negative emotion contagion from parents to adolescents by providing a relational context that promotes felt security and belonging (Chiang et al., 2025). Thus, adolescent suicidal ideation may serve as a grouping characteristic that identifies families with distinct patterns of daily emotion contagion.

Drawing on family systems theory (Cox & Paley, 2003), the bidirectional nature of family emotional systems further suggests that suicidal ideation does not operate solely as a characteristic of the adolescent that shapes parent–adolescent emotion contagion. Although no studies to date have directly examined the role of suicidal ideation in characterizing parent–adolescent emotion contagion processes, a growing body of evidence documents how adolescent psychopathology may shape dyadic emotional dynamics within the family system. Mercado et al. (2019) found that parent–youth coregulation of distress emerged only among older adolescents reporting above‐average internalizing symptoms, suggesting that adolescent psychopathology may function as a sensitizing condition that amplifies susceptibility to parental negative emotional experiences. Parents who are aware of or concerned about their adolescent's suicidal ideation may themselves experience heightened emotional insecurity, anxiety, or distress within the relationship. Research has documented that parents of adolescents with suicidal ideation commonly experience a traumatizing emotional response characterized by shock, fear, and uncertainty about how to respond to their child's psychological state (Buus et al., 2014). Research has further documented that caregiving stress among parents of psychiatrically hospitalized adolescents remains high at the time of hospitalization and that greater adolescent suicidal ideation severity in the period following discharge was associated with elevated caregiving stress over time (Micol et al., 2025). Parental emotional reorganization, driven by concern for their child's well‐being, may increase parents' sensitivity to adolescent emotional cues as they monitor their child's affective state more closely. Alternatively, parents may experience secondary dysregulation in response to the perceived threat of their child's suicidality, elevating their own negative emotional experiences within the family system. Either pathway suggests that the presence of adolescent suicidal ideation characterizes meaningful differences in the dyadic emotional environment that extend beyond the adolescent and implicate the broader relational system, consistent with family systems theory's emphasis on the interdependence of family members' psychological functioning.

An important but underexamined dimension of these processes concerns the valence‐specificity of emotion contagion in families where adolescent suicidal ideation is present. It remains unclear whether suicidal ideation primarily amplifies sensitivity to negative parental emotions, consistent with the heightened threat vigilance implied by thwarted belongingness, or whether it also disrupts responsiveness to positive parental emotions, which may be less effectively processed by adolescents in psychological pain. Similarly, at the parental level, less is known about whether parents of adolescents with suicidal ideation are more attuned to adolescent negative emotional cues, reflecting protective monitoring, or whether adolescent positive emotions may also carry heightened significance within these dyads, perhaps signaling reassurance or recovery to anxious parents. These are inherently open empirical questions that the existing literature has not addressed. The present study takes an exploratory approach to examining valence‐specific emotion contagion across both partners in dyads where adolescent suicidal ideation is present, with the goal of generating theoretically grounded insights into how suicidal ideation reorganizes the daily emotional dynamics of the parent–adolescent relationship.

#### Actor–partner interdependence model

The present study employs the actor–partner interdependence model (APIM; Kenny et al., 2006), which offers several methodological advantages over other approaches in previous studies. First, the APIM simultaneously estimates both actor effects, the association between one's own emotions and next‐day emotional experiences, and partner effects, the association between one partner's emotions and the other partner's next‐day emotional experiences, allowing for a more complete pattern of dyadic emotional processes than designs that examine parent or adolescent effects in isolation (e.g., Gadassi‐Polack et al., 2025; Yang et al., 2020). Second, by modeling next‐day emotions as outcomes, this approach captures prospective within‐dyad emotion contagion rather than concurrent associations that cannot distinguish directionality. Third, the APIM framework accommodates examination of both same‐valence contagion, such as parent negative emotions predicting adolescent negative emotions, and cross‐valence contagion, such as parent positive emotions predicting adolescent negative emotions, providing a more comprehensive account of valence‐specific processes that have been inconsistently examined in prior work. Finally, the multi‐group extension of the APIM enables direct comparison of emotion contagion patterns between adolescents with and without suicidal ideation within the same analytical framework, preserving dyadic structure while testing whether suicidal ideation characterizes the strength and directionality of daily emotional transmission between parents and adolescents.

#### The current study

The present study examined the daily associations between parent and adolescent positive and negative emotions using a daily diary design and APIM framework. Specifically, we first examined same‐valence emotion contagion, testing whether parent negative emotions were associated with greater next‐day adolescent negative emotions, and whether parent positive emotions were associated with greater next‐day adolescent positive emotions. Consistent with prior work documenting bidirectional emotional transmission in parent–adolescent dyads (Chiang et al., 2025; Mancini et al., 2016), we also examined adolescent‐to‐parent same‐valence contagion, testing whether adolescent negative and positive emotions were similarly associated with corresponding next‐day parent emotions. We hypothesized that same‐valence emotion contagion would be observed in both directions, such that higher negative emotions in one partner would be associated with greater next‐day negative emotions in the other, and higher positive emotions in one partner would be associated with greater next‐day positive emotions in the other.

Second, we examined cross‐valence emotion contagion processes, testing whether parent negative emotions were associated with lower next‐day adolescent positive emotions, and whether parent positive emotions were associated with lower next‐day adolescent negative emotions, and vice versa for adolescent‐to‐parent contagion. Cross‐valence contagion has received limited empirical attention, yet it is essential for understanding the full scope of how parental emotional experiences shape adolescent affective states across both positive and negative dimensions (Mancini et al., 2016). We hypothesized that parent negative emotions would be associated with lower next‐day adolescent positive emotions, and that parent positive emotions would be associated with lower next‐day adolescent negative emotions, reflecting the cross‐valence regulatory implications of daily parental affect.

Last, we examined daily parent–adolescent emotion contagion using multi‐group APIM analyses comparing youth with and without suicidal ideation. Drawing on the interpersonal theory of suicide (Joiner, 2005; van Orden et al., 2010) and the Three‐Step Theory (Klonsky & May, 2015), we hypothesized that families with adolescent suicidal ideation would demonstrate greater sensitivity to partner emotions relative to families without suicidal ideation, such that more contagion associations across both same‐valence and cross‐valence processes and in both parent‐to‐adolescent and adolescent‐to‐parent directions would be observed in the suicidal ideation group. Given the limited empirical basis for guiding precise directional predictions regarding valence‐specific patterns, we approached the role of suicidal ideation as exploratory, with the aim of generating theoretically grounded insights into how suicidal ideation reorganizes the daily emotional dynamics of the parent–adolescent dyad.

## METHOD

### Participants

The present study utilized data from an intensive longitudinal study examining daily family dynamics and adolescent emotional well‐being. The sample consisted of 156 adolescent‐parent dyads who completed both the baseline assessment and the daily diary component of the study. Adolescents ranged in age from 12 to 17 years (M = 14.66, SD = 1.47; 58% female), and parents ranged in age from 30 to 61 years (M = 38.66, SD = 4.68; 79% female). With respect to race and ethnicity, adolescents identified as White/European American (34.6%), Hispanic/Latinx (28.2%), African American/Black (23.1%), Asian American (11.5%), and multiracial (2.6%). Annual household income was distributed as follows: less than $19,999 (3%), $20,000 to $49,999 (11.5%), $50,000 to $89,999 (24.4%), $90,000 to $129,999 (40.5%), $130,000 to $159,999 (10.7%), $160,000 to $199,999 (2.6%), and more than $200,000 (7.3%).

### Procedure

Families were recruited through multiple channels, including community flyers, school‐based email announcements, online newsletters, social media platforms (e.g., Facebook), and referral‐based snowball sampling across Texas. Eligibility criteria required that participating families include (a) an adolescent between the ages of 12 and 17, (b) reliable internet access and a smartphone suitable for completing daily surveys, (c) English fluency for both the adolescent and caregiver, and (d) the adolescent residing with at least one primary caregiver for more than 5 days per week. Prior to enrollment, families underwent a structured screening process to verify eligibility and promote data quality, including online or in‐person interviews and cross‐checks of demographic and response consistency information. Families who successfully completed screening were enrolled in the study and completed baseline assessments before beginning the daily diary protocol. Participants received compensation of up to $90 upon study completion. Daily surveys were administered over a 14‐day period. Compliance rates were high, with adolescents completing an average of 93.08% of daily surveys and parents completing an average of 92.43%. Little's Missing Completely at Random (MCAR) test indicated that missingness patterns were consistent with an MCAR assumption, suggesting that missing data were unlikely to systematically bias within‐person estimates. The study was approved by the Institutional Review Board, and written informed consent was obtained from all participating adolescents and parents prior to enrollment.

### Measures

#### Parent daily positive and negative emotions

Parents reported their daily positive and negative emotions on 10 items adapted from the Positive and Negative Affect Schedule (PANAS; Watson et al., 1988). Using a 0–10 slider scale with a one‐unit increment (0 = not at all true, 10 = very much true), they rated five positive emotion items (e.g., happy, excited) and five negative emotion items (e.g., distressed, sad). Participants were instructed to indicate the extent to which they experienced each emotion on a given day using the following prompt: “*This scale consists of a number of words that describe different feelings and emotions. Read each item and use the slider to indicate the number from the scale below next to each word. Indicate to what extent you felt this way TODAY*.” Positive and negative items were separately averaged to create composite scores for parent positive and negative emotions. The within‐person reliability (Rc) was 0.85 for positive emotions and 0.84 for negative emotions, and the between‐person reliability (R_1F_) was 0.88 for positive emotions and 0.86 for negative emotions.

#### Adolescent daily positive and negative emotions

Adolescents also reported their daily positive and negative emotions on the same 10 PANAS‐adapted items, using a 0–10 slider scale with a one‐unit increment (0 = not at all true, 10 = very much true). They rated five positive emotion items (e.g., happy, excited) and five negative emotion items (e.g., distressed, sad), which were separately averaged to create composite scores for adolescent positive and negative emotions. The within‐person reliability (*R*c) was 0.82 for positive emotions and 0.84 for negative emotions, and the between‐person reliability (*R*_1F_) was 0.87 for positive emotions and 0.88 for negative emotions.

#### Baseline suicidal ideation

Adolescents reported suicidal ideation using a single item from the Suicidal Ideation Attributes Scale (SIDAS; Van Spijker et al., 2014): “In the past month, how often have you had thoughts about suicide?” (0 = Never, 10 = Always). Responses were used to create a dichotomous variable of suicidal ideation, with adolescents scoring 0 coded as without suicidal ideation (0) and those scoring 1–10 coded as with suicidal ideation (1). The past‐month timeframe captures recent suicidal thoughts, which is suitable for examining how baseline suicidal ideation relates to daily emotional processes over the study period.

### Analytic plan

Data analyses were conducted in R using the lavaan package (Rosseel, 2012). To examine daily parent–adolescent emotion contagion and the role of youth suicidal ideation, we employed multilevel structural equation modeling (MSEM) with the APIM (Kenny et al., 2006). The APIM treats parent–adolescent dyads as the unit of analysis, simultaneously estimating actor effects, the association between one partner's emotions and their own next‐day emotions, and partner effects, the association between one partner's emotions and the other partner's next‐day emotions. Dyad members were treated as distinguishable given the theoretical and role‐based distinctions between parents and adolescents within the family system (Kenny et al., 2006). Because we estimated fully saturated models, model fit indices are not reported.

To test the study hypotheses, we constructed a series of cross‐lagged models examining emotions at time *t* as predictors of next‐day emotions at time *t* + 1, accounting for autoregressive effects by including same‐partner emotions at time *t*. This approach allowed us to examine prospective within‐dyad emotional transmission while statistically adjusting for prior emotional states. Specifically, we first examined same‐valence emotion contagion, testing whether parent negative emotions at time t were associated with adolescent negative emotions at time *t* + 1, and whether parent positive emotions at time t were associated with adolescent positive emotions at time *t* + 1. Corresponding adolescent‐to‐parent same‐valence associations were examined simultaneously within the same model. Second, we examined cross‐valence emotion contagion, testing whether parent negative emotions at time *t* were associated with adolescent positive emotions at time *t* + 1, and whether parent positive emotions at time *t* were associated with adolescent negative emotions at time *t* + 1, with corresponding adolescent‐to‐parent cross‐valence associations estimated in the same model.

In all models, we specified correlations among same‐day emotions to account for their co‐occurrence, including correlations between parent positive and negative emotions at time *t*, between adolescent positive and negative emotions at time *t*, and between parent and adolescent emotions at time *t*. Similarly, residual correlations among next‐day emotions at time *t* + 1 were specified to account for shared variance not explained by the cross‐lagged paths.

Last, to examine the role of youth suicidal ideation, we conducted multi‐group APIM analyses comparing parent–adolescent dyads with and without adolescent suicidal ideation. This approach retained the dyadic structure of the data while enabling direct comparison of actor and partner effects across groups, allowing us to test whether suicidal ideation was associated with differential patterns of same‐valence and cross‐valence emotion contagion in both parent‐to‐adolescent and adolescent‐to‐parent directions. All models controlled for parent gender, adolescent gender, family income, and adolescent race and ethnicity as covariates.

## RESULTS

Table 1 showed the descriptive statistics and correlations. At the within‐family level, parent negative emotions were negatively correlated with parent positive emotions (*r* = −0.51, *p* < .001), positively correlated with youth negative emotions (*r* = 0.29, *p* < .001), and negatively correlated with youth positive emotions (*r* = −0.04, *p* < .01). Parent positive emotions were negatively correlated with youth negative emotions (*r* = −0.19, *p* < .001) but not correlated with youth positive emotions (*r* = 0.02, *p* > .05). Youth negative emotions were negatively correlated with youth positive emotions (*r* = −0.39, *p* < .001).

**TABLE 1:** Descriptive statistics and correlations.

	1.	2.	3.	4.	5.
**Within‐family level**					
1. Parent negative emotions	–				
2. Parent positive emotions	−0.51***	–			
3. Youth negative emotions	0.29***	−0.19***	–		
4. Youth positive emotions	−0.04**	0.02	−0.39***	–	
**Between‐family level**					
1. Parent negative emotions	–				
2. Parent positive emotions	−0.31***	–			
3. Youth negative emotions	0.18*	−0.07	–		
4. Youth positive emotions	−0.01	0.00	−0.24**	–	
5. Suicidal ideation	0.06	−0.08	0.16*	−0.17*	–
Mean	1.32	7.14	1.68	6.70	0.34
SD	1.78	2.12	1.45	2.43	0.46

*p < .05; **p < .01; ***p < .001.

**FIGURE 1:**
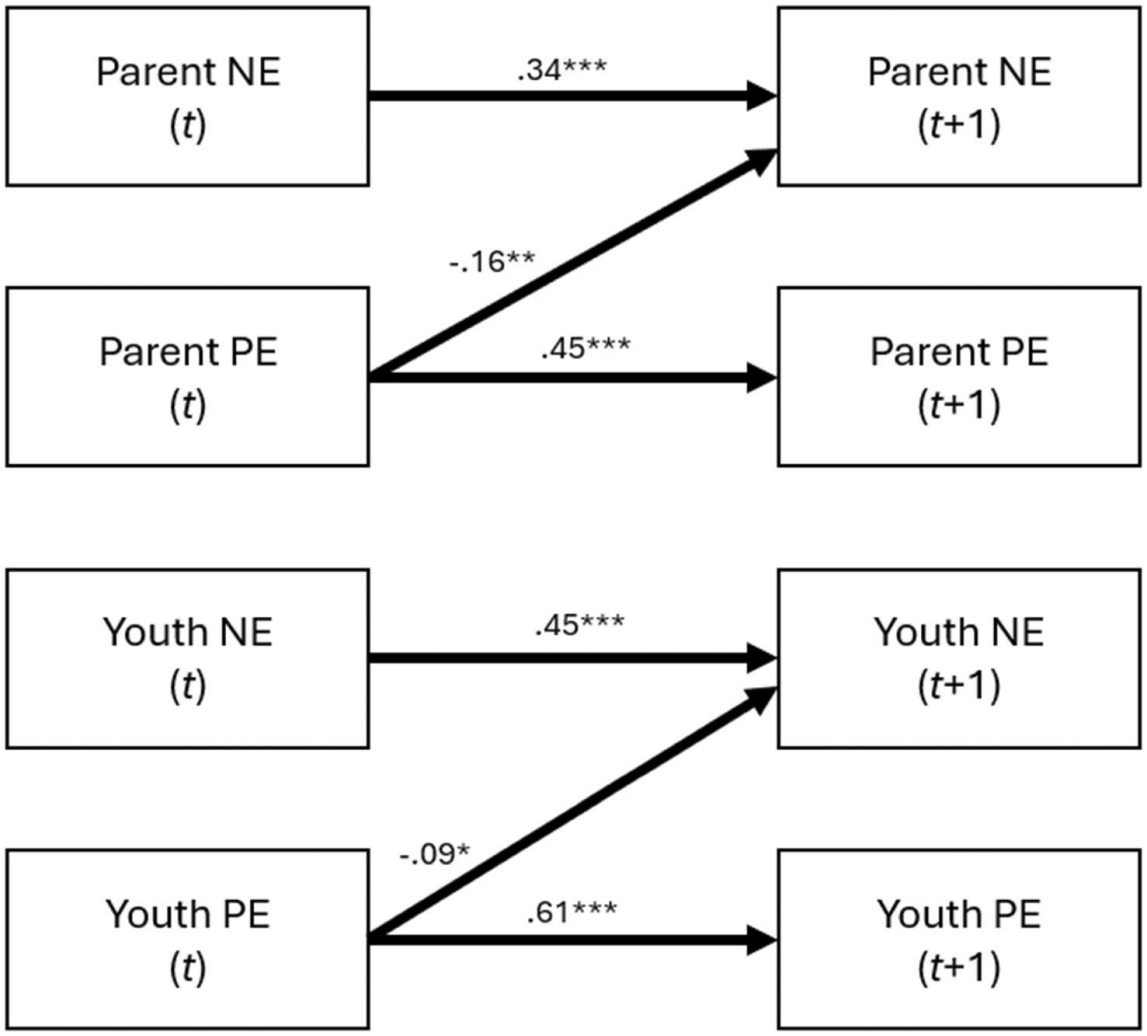
Associations between parent–adolescent emotion contagion. *p < .05; **p < .01; ***p < .001. All same‐day correlations were specified. Black lines indicate actor effects (i.e., parent emotion paths).

As shown in Figure 1, results from the APIM analysis revealed that higher parent negative emotions were associated with higher next‐day negative emotions (*β* = .34, *p* < .001), while parent positive emotions were associated with lower next‐day negative emotions (β = −.16, *p* < .01) and higher next‐day positive emotions (*β* = .45, *p* < .001). Moreover, higher youth negative emotions were associated with higher next‐day negative emotions (*β* = .45, *p* < .001), while youth positive emotions were associated with lower next‐day negative emotions (*β* = −.09, *p* < .05) and higher next‐day positive emotions (*β* = .61, *p* < .001). Together, these findings indicate that both parent and adolescent daily emotions prospectively predicted their own next‐day emotional experiences. We also reported same‐day correlations between parent and youth emotions in Table 2.

**TABLE 2:** Within‐day correlations of multi‐group analyses.

	Day (*t*)	Day (*t* + 1)
	*β*	*β*
Main results		
Parent NE ↔ Parent PE	−.51***	−.39***
Parent PE ↔ Youth NE	−.18**	−.16*
Youth NE ↔ Youth PE	−.37***	−.39***
Parent NE ↔ Youth NE	.29***	.22***
Parent NE ↔ Youth PE	−.04	−.11*
Parent PE ↔ Youth PE	.02	.14**
Multi‐group results (without/with SI)		
Parent NE ↔ Parent PE	−.54***/−.38*	−.47***/−.23*
Parent PE ↔ Youth NE	−.18*/.03	−.23*/−.01
Youth NE ↔ Youth PE	−.40*/−.13	−.47***/−.21**
Parent NE ↔ Youth NE	.28**/.18	.25**/.15*
Parent NE ↔ Youth PE	−.06/.17	−.13/−.01
Parent PE ↔ Youth PE	.03/−.26	.13*/.10

*Note*: SI indicates suicidal ideation.

*p < .05; **p < .01; ***p < .001.

**FIGURE 2:**
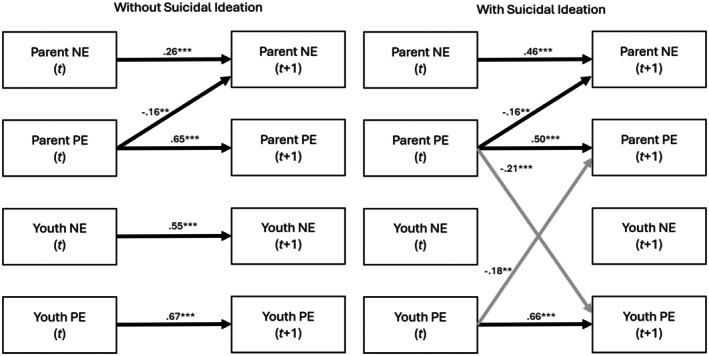
Results of multi‐group APIM models of parent–adolescent emotion contagion between youth with and without suicidal ideation. **p < .01; ***p < .001. All same‐day correlations were specified. Black lines indicate actor effects (i.e., parent emotion paths). Gray lines indicate partner effects (i.e., parent‐to‐youth or youth‐to‐parent paths).

To examine whether these associations differed as a function of adolescent suicidal ideation, we conducted multi‐group APIM analyses comparing dyads with and without adolescent suicidal ideation. Results are presented in Figure 2. Among adolescents without suicidal ideation, findings were consistent with the overall model. Higher parent negative emotions were associated with greater next‐day parent negative emotions (*β* = .26, *p* < .001), while parent positive emotions were associated with lower parent next‐day negative emotions (*β* = −.16, *p* < .01) and greater next‐day positive emotions (*β* = .65, *p* < .001). Higher youth negative emotions were associated with greater next‐day youth negative emotions (*β* = .55, *p* < .001), while youth positive emotions were associated with greater next‐day youth positive emotions (*β* = .67, *p* < .001). In contrast, the findings were different in the group of youth with suicidal ideation. Higher parent negative emotions were associated with higher next‐day negative emotions (*β* = .46, *p* < .001), while parent positive emotions were associated with lower next‐day negative emotions (*β* = −.16, *p* < .01) and higher next‐day positive emotions (*β* = .50, *p* < .001). Furthermore, parent positive emotions were also associated with lower next‐day youth positive emotions (*β* = −.21, *p* < .001). Higher youth positive emotions were associated with higher next‐day positive emotions (*β* = .66, *p* < .001), as well as lower next‐day parent positive emotions (*β* = −.18, *p* < .01). Last, we examined the correlations between same‐day parent and youth emotions and found there were two differences between the groups. In the group of youth without suicidal ideation, parent positive emotions were associated with lower youth negative emotions (*β* = −.18, *p* < .05), and parent negative emotions were associated with greater youth negative emotions (*β* = .28, *p* < .01). However, these associations were not significant in the group of youth with suicidal ideation.

To test whether the observed group differences were statistically significant, we conducted individual path constraint tests focusing on the two partner effects that emerged in the suicidal ideation group. Each path was constrained to equality across groups one at a time, and the fit of each model was compared to the freely estimated multi‐group model. Results indicated that each individually constrained path resulted in a significantly worse model fit relative to the freely estimated model (Δ*χ* ^2^ (1) = 4.58 and 6.62, *p*s < .05), providing statistical evidence that these emotion contagion pathways differed significantly between families with and without adolescent suicidal ideation.

Moreover, we conducted complementary analyses to explore the transactional relationship between daily emotion contagion and adolescent suicidal ideation using Mplus 8.11. Specifically, between‐family random slopes for the prospective partner effect of parent negative emotions on next‐day adolescent negative emotions were estimated within the MSEM framework, and these slopes were subsequently used to predict the likelihood of membership in the suicidal ideation group. One significant association was identified: results indicated that greater negative emotion contagion from parents to adolescents was associated with higher likelihood of adolescent suicidal ideation group membership, suggesting that families characterized by stronger negative emotion contagion from parents to adolescents were more likely to include an adolescent reporting suicidal ideation.

In addition, supplementary analyses examined whether the observed associations differed as a function of parent and adolescent gender. Parent gender and adolescent gender were tested as moderators of the main APIM pathways and the multi‐group associations. No significant gender differences emerged in either the primary analyses or the multi‐group models, suggesting that no gender differences were found in the observed patterns of emotion contagion and the role of adolescent suicidal ideation in these associations.

## DISCUSSION

The present study examined daily parent–adolescent emotion contagion and the role of adolescent suicidal ideation using a daily diary design and multi‐group APIM framework. Findings suggest that both parents and adolescents showed prospective within‐person emotional continuity across consecutive days, with daily emotions predicting their own next‐day emotional experiences across same‐valence and cross‐valence pathways. However, limited parent‐to‐youth or youth‐to‐parent emotion contagion was observed. With respect to the role of suicidal ideation, families with adolescent suicidal ideation demonstrated distinct patterns of dyadic emotion contagion that were absent among families without suicidal ideation, particularly the novel partner effects involving positive emotions in both parent‐to‐adolescent and adolescent‐to‐parent directions. Together, these findings extend the emotion contagion literature by documenting valence‐specific and temporally structured patterns of daily emotional transmission in parent–adolescent dyads, and by providing initial evidence that adolescent suicidal ideation shapes the dyadic emotional experiences in ways that implicate both partners.

Consistent with prior daily diary research documenting within‐person emotion contagion (Larson & Gillman, 1999; Moed et al., 2017), actor effects were observed for both parents and adolescents across same‐valence and cross‐valence pathways, indicating that daily emotional experiences prospectively shaped next‐day emotional states within each individual. However, the primary focus of the present study concerned partner effects, that is, whether one partner's daily emotions were associated with the other partner's next‐day emotional experiences. In the overall sample, partner effects between parents and adolescents were limited, with no significant prospective associations emerging from parent to adolescent or from adolescent to parent. This pattern of limited partner effects may reflect several explanations. First, emotion contagion processes may be more likely to unfold within shorter time frames within the same day rather than extending across days following sleep, suggesting that the present study's focus on prospective next‐day associations represents a more conservative test of emotion contagion that may not fully capture the more immediate and temporally proximal processes through which daily emotions are transmitted between parents and adolescents. Second, the limited partner effects may also reflect the more conservative analytical approach employed in the present study. Unlike prior work that examined same‐valence contagion in isolation (e.g., Mercado et al., 2019) or cross‐valence contagion separately from same‐valence processes (e.g., Mancini et al., 2016), the present study simultaneously modeled both same‐valence and cross‐valence pathways within the same APIM framework while also accounting for same‐day emotional associations between partners. This approach increases the robustness of each individual path estimate by accounting for the shared variance across valence‐specific contagion processes as well as within‐day emotional coupling, which may have attenuated the detection of individual partner effects that would otherwise reach significance when modeled in isolation. Importantly, the same‐day emotional associations documented in the present study are themselves meaningful indicators of emotion contagion and are discussed as substantive findings in subsequent sections. This methodological distinction underscores the value of comprehensive temporal modeling for understanding the full scope of daily emotion contagion in parent–adolescent dyads.

The multi‐group APIM results showed that parent–adolescent emotion contagion was more evident among youth with suicidal ideation relative to youth without suicidal ideation. Among families with adolescent suicidal ideation, two novel partner effects emerged that were absent in families without suicidal ideation, both involving positive emotions and operating in a negative direction. Specifically, higher parent positive emotions were associated with lower next‐day adolescent positive emotions, and higher adolescent positive emotions were associated with lower next‐day parent positive emotions. This bidirectional pattern of inverse partner effects involving positive emotions represents the most theoretically distinctive finding of the present study and warrants careful interpretation.

From the perspective of the interpersonal theory of suicide (Joiner, 2005; van Orden et al., 2010), adolescents experiencing thwarted belongingness and perceived burdensomeness maintain a state of chronic interpersonal hypervigilance that may fundamentally alter how parental emotional cues are processed. This relational sensitivity may be further understood through Linehan's (1993) biosocial theory, which posits that individuals with heightened emotional vulnerability who develop within invalidating family environments fail to acquire adaptive skills for labeling, tolerating, and regulating emotional experiences. An invalidating environment is one in which a parent's emotional expression is discrepant from or dismissive of the adolescent's own emotional state, communicating implicitly that the adolescent's emotional experience is unwarranted or incorrect (Crowell et al., 2009). For adolescents already experiencing suicidal ideation, encounters with parental positive emotions may function as a form of emotional invalidation, insofar as the parent's positive affective state is fundamentally discrepant from the adolescent's own psychological pain. Rather than facilitating emotional coregulation, such discrepancy may reinforce the adolescent's sense that their internal experience is misunderstood or invisible within the relationship, thereby suppressing rather than elevating their positive emotions the following day.

Emerging neurobiological evidence is consistent with this view, suggesting that youth with suicidal ideation demonstrate blunted subcortical responses to positive social stimuli, including attenuated hippocampal and amygdala activation in response to happy faces (Quevedo et al., 2016), indicating that the affiliative and reward‐signaling functions of positive emotional expression may be neurobiologically disrupted. At the psychological level, adolescents experiencing suicidal ideation may operate with a chronically lowered threshold for interpersonal distress (Miller & Prinstein, 2019), such that a parent's positive emotional expression amplifies rather than alleviates the adolescent's sense of disconnection. When a parent expresses positive emotions, rather than experiencing these displays as affirming or connecting, an adolescent in psychological pain may experience a heightened sense of contrast between the parent's positive state and their own distress, potentially intensifying feelings of burdensomeness within the relationship. Other studies related to youth psychological problems could also support this. Adolescents with elevated depressive symptoms were more likely to interpret ambiguous situations as negative rather than positive, leading to both interpretation and attention bias toward neutral or positive scenarios (e.g., Klein et al., 2018; Orchard et al., 2016). This contrast effect, wherein positive interpersonal signals compound rather than alleviate psychological pain, is consistent with the Three‐Step Theory's proposition that connectedness operates differently for individuals already experiencing significant psychological distress (Klonsky & May, 2015). As a result, parent positive emotions may inadvertently function as a dysregulating rather than buffering interpersonal input for adolescents with suicidal ideation, suppressing rather than elevating their positive affect the following day.

The reverse pattern, wherein adolescent positive emotions predicted lower parent positive emotions the following day, is equally theoretically important and can be interpreted through the lens of family systems theory (Cox & Paley, 2003). Parents of adolescents with suicidal ideation who observe their child displaying positive emotions may not experience these displays as straightforwardly reassuring. Rather, parental hypervigilance about the stability and authenticity of the adolescent's positive affect may generate uncertainty or anxiety about whether the positive state reflects genuine improvement or a temporary and potentially unstable fluctuation. This type of parental guardedness is consistent with qualitative evidence documenting that parents of youth with suicidal ideation characteristically respond with emotional distancing and disrupted empathic capacity when processing their child's emotional states (Lachal et al., 2015), and that even in structured clinical contexts parents often struggle to receive their child's behavior as meaningfully indicative of improvement (Slovak & Singer, 2012). The adverse effects of suicidal ideation within the family, as Lachal et al. (2015) describe, may produce a chronic relational disruption in which parents remain emotionally guarded and unable to allow the adolescent's positive affect to be interpreted as genuine or stable. As such, the interpretive ambiguity surrounding the adolescent's positive emotional displays may suppress parent positive emotions the following day, as parents remain emotionally guarded rather than allowing the adolescent's positive emotions to elevate their own affective state. Consistent with the TIES framework (Butler, 2011), these patterns suggest that in dyads with suicidal ideation the functions of positive emotion expression within the dyad have become dysregulated, such that positive emotional signals from either partner fail to elicit reciprocal positive emotions and may instead introduce emotional uncertainty that dampens the receiving partner's next‐day positive experience.

Regarding same‐day associations between parent and youth emotions, a distinct pattern emerged across groups with respect to the coupling between parent and adolescent emotions within the same day. Among families without adolescent suicidal ideation, parent positive emotions were concurrently associated with lower adolescent negative emotions, and parent negative emotions were concurrently associated with greater adolescent negative emotions, suggesting that parental emotional states were linked to adolescent emotional experiences within the same day. This pattern is consistent with prior research documenting immediate within‐day emotional transmission in parent–adolescent dyads (Chiang et al., 2025; Larson & Gillman, 1999; Mercado et al., 2019). Notably, neither of these same‐day associations reached significance in the suicidal ideation group, suggesting that the immediate emotion contagion between parents and adolescents that characterizes typical family functioning may be attenuated or absent in families where adolescents are experiencing suicidal ideation. Rather than reflecting immediate same‐day emotional responsiveness, the suicidal ideation group demonstrated the prospective partner effects described above, suggesting a possible shift in the temporal structure of dyadic emotion contagion, from concurrent to delayed, in families with elevated suicide risk.

The present findings also have critical implications for clinical practice and family‐based suicide prevention efforts. Existing interventions for adolescent suicidal ideation often emphasize improving family communication, emotional validation, and parent–adolescent connectedness (Asarnow et al., 2017; Diamond et al., 2019). The current findings suggest that clinicians may benefit from paying particular attention not only to the presence of negative emotions within families but also to how positive emotions are expressed, interpreted, and received during parent–adolescent interactions. Among families with adolescent suicidal ideation, positive emotional experiences appeared to function differently than would typically be expected, with positive emotions in one partner predicting lower positive emotions in the other partner on the following day. This pattern may indicate that positive emotional expressions are not consistently experienced as reassuring, validating, or relationship‐enhancing within these dyads. Clinically, these findings highlight the potential value of helping parents and adolescents develop greater awareness of how emotions are communicated and interpreted, particularly during periods of elevated psychological distress. Interventions that strengthen emotional validation, increase mutual understanding of emotional experiences, and promote adaptive dyadic coregulation may help restore the affiliative and supportive functions of positive emotional exchanges within families of adolescents experiencing suicidal ideation.

Several limitations of the present study warrant consideration. First, our data were based on self‐report daily diary measures, which are subject to reporting biases and may not fully capture the complexity of emotional experiences as they unfold in daily family life. Although ecological momentary assessment approaches such as daily diary designs are well‐suited to capturing within‐person variability in naturalistic contexts (Bolger & Laurenceau, 2013), the reliance on self‐rating limits the depth of assessment relative to multi‐dimensional, naturalistic measures such as video‐coded facial expressions of emotions and emotion elicitation in a laboratory (Lench et al., 2011; McNeil & Repetti, 2021). Second, suicidal ideation was assessed using a single baseline item and dichotomized for the purpose of multi‐group comparison. This approach, while pragmatically justified given the daily diary context and the need to preserve dyadic sample size across groups, necessarily obscures variation in the severity, chronicity, and clinical salience of suicidal ideation within the suicidal ideation group. Future research would benefit from incorporating validated multi‐item measures of suicidal ideation to permit more nuanced evaluation of suicide risk. Third, the present study did not include clinical diagnostic data, and it is therefore not possible to determine the extent to which the observed group differences reflect suicidal ideation specifically, as opposed to co‐occurring internalizing psychopathology such as depression or anxiety that frequently co‐occurs with suicidal ideation in adolescent samples (Nock et al., 2013). Future research incorporating structured diagnostic interviews would allow for more precise isolation of suicidal ideation as a sensitizing process independent of broader internalizing symptomatology. Fourth, the present sample was drawn from a single geographic region and was predominantly comprised of families with middle‐range household incomes, which may limit the generalizability of findings to families from different socioeconomic, cultural, or geographic contexts. Finally, the present study examined dyadic emotion contagion exclusively within parent–adolescent dyads and did not account for broader family system dynamics, including the potential role of sibling relationships, marital quality, or broader household emotional climate, each of which may shape the daily emotional transmission processes documented.

In conclusion, the present study examined daily parent–adolescent emotion contagion and the role of adolescent suicidal ideation using a 14‐day daily diary design and a multi‐group APIM framework. In families without adolescent suicidal ideation, same‐day emotional coupling between parent and adolescent negative emotions was observed, consistent with prior studies of immediate within‐day emotion contagion in parent–adolescent dyads. Families with adolescent suicidal ideation demonstrated a unique, distinct pattern, characterized by inverse prospective emotion contagion in which parent and adolescent positive emotions each predicted lower positive emotions in the other partner the following day, alongside an absence of same‐day emotion contagion between partners. These findings suggest that adolescent suicidal ideation is associated with a fundamental reorganization of dyadic emotional functioning in which the normal affiliative functions of positive emotion contagion are disrupted. Family‐based prevention and intervention efforts may therefore benefit from explicitly targeting positive emotional communication and dyadic coregulation skills, recognizing that the restoration of healthy emotion contagion between parents and adolescents represents a meaningful but understudied target of suicide prevention and family mental health promotion.

## AUTHOR CONTRIBUTIONS

**Shou‐Chun Chiang:** Conceptualization; data curation; formal analysis; writing – original draft; writing – review and editing.

## FUNDING INFORMATION

The author has nothing to report.

## CONFLICT OF INTEREST STATEMENT

The author declares no conflict of interest.

## ETHICS STATEMENT

The study was approved and followed by the Institutional Review Board of Texas Tech University (IRB2024‐949) on December 16, 2024. The study is in accordance with the Declaration of Helsinki.

## CONSENT

Informed consent was obtained from all individual participants included in the study.

## Data Availability

The data that support the findings of this study are available from the corresponding author upon reasonable request.
